# Second WIN International Conference on “Integrated approaches and innovative tools for combating insecticide resistance in vectors of arboviruses”, October 2018, Singapore

**DOI:** 10.1186/s13071-019-3591-8

**Published:** 2019-07-03

**Authors:** Vincent Corbel, Claire Durot, Nicole L. Achee, Fabrice Chandre, Mamadou B. Coulibaly, Jean-Philippe David, Gregor J. Devine, Isabelle Dusfour, Dina M. Fonseca, John Griego, Waraporn Juntarajumnong, Audrey Lenhart, Shinji Kasai, Ademir J. Martins, Catherine Moyes, Lee Ching Ng, João Pinto, Julien F. Pompon, Pie Muller, Kamaraju Raghavendra, David Roiz, Hassan Vatandoost, John Vontas, David Weetman

**Affiliations:** 1 Institut de Recherche pour le Développement (IRD), Maladies Infectieuses et Vecteurs, Ecologie, Génétique, Evolution et Contrôle (MIVEGEC UM1-CNRS 5290-IRD 224), B.P. 64501, 911 Avenue Agropolis, 34394 Montpellier Cedex 5, France; 2 Department of Biological Sciences, Eck Institute for Global Health, University of Notre Dame (UND), 239 Galvin Life Science Center, Notre Dame, IN 46556 USA; 3 Malaria Research and Training Center (MRTC), Point G, B.P. 1805, Bamako, Mali; 4 Laboratoire d’Ecologie Alpine (LECA), Centre National de la Recherche Scientifique (CNRS), UMR 5553, CNRS, Université Grenoble-Alpes, Domaine universitaire de Saint-Martin d’Hères, 2233 rue de la piscine, 38041 Grenoble Cedex 9, France; 5 Mosquito Control Laboratory, QIMR Berghofer Medical Research Institute, 300 Herston Road, Herston, Queensland 4006 Australia; 6 Institut Pasteur de la Guyane (IPG), 23 avenue Pasteur B.P. 6010, 97306 Cayenne Cedex, French Guiana; 7 Rutgers University (RU), Center for Vector Biology, 180 Jones Avenue, New Brunswick, NJ 08901 USA; 8 Department of Entomology, Kasetsart University (KU), 50 Ngam Wong Wan Rd, Ladyaow Chatuchak, Bangkok, 10900 Thailand; 9 Center for Global Health/Division of Parasitic Diseases and Malaria/Entomology Branch, U.S. Centers for Disease Control and Prevention (CDC), 1600 Clifton Rd. NE, MS G-49; Bldg. 23, Atlanta, GA 30329 USA; 10 Department of Medical Entomology, National Institute of Infectious Diseases, 1-23-1 Toyama, Shinjukuku, Tokyo, Japan; 11 Instituto Oswaldo Cruz (Fiocruz), Avenida Brasil, 4365, Manguinhos, Rio de Janeiro, RJ CEP: 21040-360 Brazil; 12 Big Data Institute, Li Ka Shing Centre for Health Information and Discovery, University of Oxford, Oxford, OX3 7LF UK; 13 Environmental Health Institute (EHI), National Environment Agency (NEA), 11 Biopolis Way, Helios Block, #04-03/04 & #06-05/08, Singapore, Singapore; 14 Global Health and Tropical Medicine, GHTM, Instituto de Higiene e Medicina Tropical, IHMT, Universidade Nova de Lisboa, UNL, Rua da Junqueira 100, 1349-008 Lisboa, Portugal; 15 Programme in Emerging Infectious Diseases, Duke-NUS Medical School, Singapore, 169857 Singapore; 16 Department of Epidemiology and Public Health, Swiss Tropical and Public Health Institute, Socinstrasse 57, PO Box 4002, Basel, Switzerland; 17 University of Basel, Petersplatz 1, 4001 Basel, Switzerland; 18 Department of Health Research, ICMR-National Institute of Malaria Research (NIMR), GoI Sector 8, Dwarka, Delhi 110 077 India; 19 Department of Medical Entomology & Vector Control, Tehran University of Medical Sciences (TUMS), School of Public Health and Institute for Environmental Research, Pour Sina Street, P.O. Box: 14155-6446, Tehran, Iran; 20 Institute Molecular Biology and Biotechnology (IMBB), Foundation for Research and Technology (FORTH), Panepistimioupoli, Voutes, 70013 Heraklio, Crete Greece; 21 Pesticide Science Laboratory, Agricultural University of Athens, Ieara Odoes 75, 118 Athens, Greece; 22 Department of Vector Biology, Liverpool School of Tropical Medicine (LSTM), Pembroke Place, Liverpool, L35QA UK

**Keywords:** Arbovirus, Mosquito, Insecticide resistance, Vector control, WIN network, Surveillance, Standardization, Strategic planning, Innovative tools

## Abstract

The past 40 years have seen a dramatic emergence of epidemic arboviral diseases transmitted primarily by mosquitoes. The frequency and magnitude of the epidemics, especially those transmitted by urban *Aedes* species, have progressively increased over time, accelerating in the past 10 years. To reduce the burden and threat of vector-borne diseases, the World Health Organization (WHO) has recently adopted the Global Vector Control Response (GVCR) in order to support countries in implementing effective sustainable vector control. The evidence-base to support vector control is however limited for arboviral diseases which make prioritization difficult. Knowledge gaps in the distribution, mechanisms and impact of insecticide resistance on vector control impedes the implementation of locally tailored *Aedes* control measures. This report summarizes the main outputs of the second international conference of the Worldwide Insecticide resistance Network (WIN) on “*Integrated approaches and innovative tools for combating insecticide resistance in arbovirus vectors*” held in Singapore, 1–3 October 2018. The aims of the conference were to review progress and achievements made in insecticide resistance surveillance worldwide, and to discuss the potential of integrated vector management and innovative technologies for efficiently controlling arboviral diseases. The conference brought together 150 participants from 26 countries.

## Background

Arboviruses transmitted by *Aedes* mosquitoes such as dengue, Zika, chikungunya, yellow fever, and recently Mayaro virus represent an increasing threat to public health worldwide [1]. The Global Vector Control Response (GVCR) recently adopted by the WHO assembly aims to reduce the burden and threat of vector-borne diseases by 2030 through effective, locally-adapted sustainable vector control [2]. The evidence-base to support vector control is limited for arboviral diseases (ABVs) due to a lack of research support and intervention data, especially in areas where mosquitoes are resistant to commonly used public health pesticides [3].

A recent systematic review [4] highlights that 57 countries already reported resistance or suspected resistance to at least one chemical class of insecticides in *Aedes aegypti* or *Ae. albopictus* mosquitoes. Resistance is now recognized as a major threat for the control of ABVs and has likely contributed to their re-emergence and spread in some parts of the world [5]. Important knowledge gaps remain on mosquito resistance including its distribution, dynamics, mechanisms, fitness costs and its impact on vector control efficacy [4]. Furthermore, there is an urgent need to review progress and achievements made in the deployment of integrated approaches and innovative technologies for the surveillance and control of arbovirus vectors [3] and to discuss their potential for mitigating insecticide resistance [6].

In March 2016, TDR, the Special Programme for Research and Training in Tropical Diseases, in collaboration with the WHO Neglected Tropical Diseases Department (NTD/WHO), supported the launch of the first-ever international network to track insecticide resistance in mosquito vectors of arboviruses. The Worldwide Insecticide Resistance Network (WIN) (http://win-network.ird.fr/), aims to enhance the surveillance of insecticide resistance worldwide, filling knowledge gaps and guiding decision making for improved insecticide resistance management strategies and vector control [7].

From 1 to 3 October 2018, the WIN organized its 2nd International Conference on “*Integrated approaches and innovative tools for combating insecticide resistance in arbovirus vectors*”. Held in Singapore, the conference was organized jointly by the French Institut de Recherche pour le Développement (IRD) and Duke-NUS Medical School of Singapore and has been recognized as an event of the “France-Singapore Year of Innovation 2018”. The first WIN international conference, held in Rio de Janeiro, Brazil from 5 to 8 December 2016, highlighted the need for more partnerships between academia, research institutions, international organizations, stakeholders, the civil society and the private sector to manage insecticide resistance and sustain vector control in endemic areas and countries facing vector-borne disease outbreaks [8]. Consequently, during the 2018 conference, representatives from 69 institutions working on vector-borne diseases were present including research institutions and universities, WHO, ministries of health, environment, foreign affairs and defense, but also members of the private sectors. This multi-sectorial conference brought together about 150 participants from 26 nationalities.

During this second conference, three scientific plenary sessions were organized: the first session dedicated to the “*Control of emerging arboviral diseases*” addressed the public health priorities and responses for reducing the burden of arboviral diseases. The second session was dedicated to “*Insecticide resistance*” and focused on the levels, spatial distribution, mechanisms and impact of insecticide resistance on arbovirus control and resistance management options. The last plenary session was dedicated to “*Innovative vector control approaches*” and presented community-based and integrated approaches for *Aedes* mosquito control and discussed the latest developments (chemical, biological and genetic tools) for reducing arbovirus transmission. Each plenary session comprised multiple presentations by scientists followed by open discussions with all participants. Scientific sessions were followed by a plenary “*Public-private initiatives in public health*”, where representatives of the agrochemical sector, research institutions, vector control consortium and international organizations presented initiatives for fostering innovation in public health. In addition, 25 posters were presented by scientists and industry. Finally, two round tables open to all participants were organized to leverage the knowledge of the audience into strategies that may accelerate the translation of vector research into policies and programmes. The meeting agenda, list of speakers, registered participants and presentations are available at https://WINSingapore2018.com.

## Welcoming addresses

The first day was opened with welcoming addresses by representatives of the National Environment Agency of Singapore (NEA), the French Ministry of Foreign Affairs in Singapore, the Duke-NUS Medical School of Singapore, and the WHO NTD and TDR departments. All speakers acknowledged the need to improve the surveillance and control of arbovirus vectors that also requires knowledge of the mosquitoes’ insecticide resistance status so that we will be better prepared against existing and emerging *Aedes*-borne disease threats. Dr Julien Pompon (Duke-NUS) welcomed participants and presented the objectives of the conference. Finally, Dr Vincent Corbel (IRD, France) thanked all sponsors, partners, and supporting organizations that contributed to the organization of the conference.

## Session 1: Control of emerging arboviral diseases

Dr Duane Gubler (Duke-NUS Medical School, Singapore) opened the first session by reviewing the changing epidemiology of potentially epidemic ABVs and the prospects for prevention and control. Beyond dengue, chikungunya and Zika, a number of viruses are circulating such as Japanese encephalitis, Ross River, Rift Valley fever, West Nile virus and others. Increased urbanization, demographic changes, increasing transportation (4 billion passengers are estimated to have traveled by air in 2018), and lack of effective vector control have greatly facilitated the movement of these viruses around the world [9]. Pandemic yellow fever (YFV) is now seen as the next public health threat, as the numbers of cases in urban settings have drastically increased in recent years [10, 11]. Despite cases being transported around the world (with particular concern in the Americas and Asia) no local transmission has occurred to date beyond Africa. Risk factors for YFV expansion are the low herd immunity in humans, encroachment of humans on sylvatic cycle, population movement, inadequate vaccine supply, and ineffective vector control. As the chairman of the Global Dengue & *Aedes*-Transmitted Diseases Consortium (GDAC), the speaker concluded that the risk of epidemic ABVs is the highest in history and encouraged the development of a “Global Fund” for ABVs in order to build in-country capacity to respond more effectively to these threats.

Dr Raman Velayudhan (Neglected Tropical Diseases Department, World Health Organization, Switzerland), presented the WHO Global Vector Control Response (GVCR), which aims to reduce the threat of vector-borne diseases through effective locally-adapted vector control strategies [2]. The success of this strategy relies on the ability of countries to strengthen their vector surveillance and control programmes with enhanced capacity and financial resources. The GVCR strategies need to focus on the following key areas: (i) aligning actions across sectors, such as ministries of health and other relevant ministries and city planners, e.g. for removing urban breeding sites; (ii) engaging local communities to protect themselves and build resilience against future disease outbreaks; (iii) strengthening surveillance to trigger early responses and to identify when and why interventions are not working as expected; and (iv) scaling-up vector-control tools and using them in combination to maximize impact on disease. The overall emphasis of this programme is to fulfill country and regional needs by strengthening vector control programmes through training and capacity building.

**Fig. 1:**
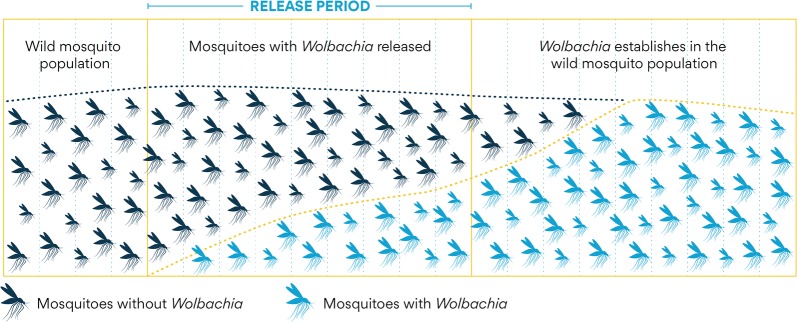
Concept of the population replacement strategy using the *wMel* strain of *Wolbachia* (Courtesy of the World Mosquito Programme)

Dr Scott O’Neill (Monash University, Vietnam) provided an update on the use of the intracellular bacteria *Wolbachia* by the World Mosquito Programme (WMP) to disrupt dengue, Zika and chikungunya transmission by *Aedes aegypti* without the need to suppress the mosquito population. The aim of the WMP is to introduce *Wolbachia*-infected mosquitoes (male and female) into wild mosquito populations to increase the frequency of *Wolbachia* carrying mosquitoes and hence to interrupt disease transmission (known as a population replacement strategy) (Fig. 1). Deployment of *Wolbachia* into *Ae. aegypti* populations can be scaled to areas around 100 km^2^ by releasing approximately 2–5 mosquitoes per person per week [12]. Pilot studies conducted in Townsville and Cairns, Australia (in 2005) showed > 90% of locally acquired dengue cases after the release of *Wolbachia*. The WMP has developed methods for low-cost, large-scale application across urban areas in countries affected by mosquito-borne diseases. The programme is now conducting efficacy trials in 11 countries (including Sri Lanka, India, Vietnam, Indonesia, Kribati, Vanuatu, Fiji, New Caledonia, Mexico, Colombia and Brazil) in order to generate data in various epidemiological settings.

Dr Didier Fontenille (Institut Pasteur, Cambodia) gave a talk entitled “*Arbovirus vectors in South East Asia: a plea of ignorance*”. Numerous factors such as deforestation/reforestation, climate change, urbanization, land use, pesticide use and human behavior contribute to the transmission risk. In Southeast Asia, particularly Cambodia, numerous gaps remain in our knowledge of the biology and ecology of arbovirus vectors and those gaps will remain as long as there is failure to develop the local research facilities and capacities required to address that goal. The recent introduction of *Ae. albopictus* in several locations of Phnom Penh and the increasing resistance of *Ae. aegypti* to public health pesticides may compromise vector control efforts. More than 6000 dengue cases were declared in Cambodia in 2018. Yellow fever is on the rise and the risk of autochthonous transmission in Asia-Pacific region has never been so high [13]. Community-based participation (COMBI) and innovative tools (traps, genetically-modified mosquitoes, *Wolbachia*) are urgently needed to improve the control of invasive mosquitoes and to prevent new arbovirus epidemics in the region.

**Fig. 2:**
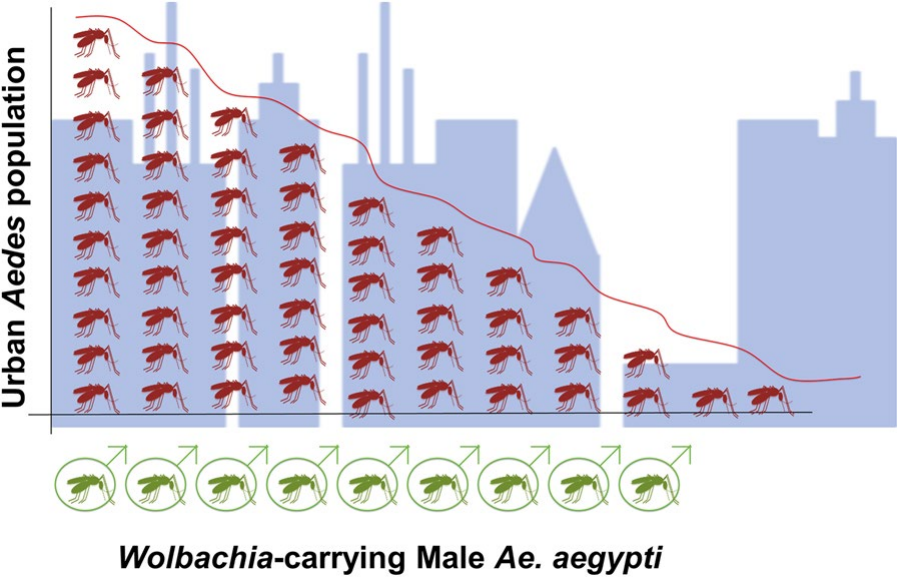
Concept of the *Wolbachia* population suppression through cytoplasmic incompatibility (Courtesy of Dr L.C. Ng, NEA, Singapore)

Professor Lee Ching Ng (National Environment Agency, Singapore) presented progress and limitations during the implementation of the “*Wolbachia* Singapore” project. The National Environment Agency (NEA) is evaluating the use of *Wolbachia*-infected *Ae. aegypti* males with the aim of breaking the dengue transmission through vector suppression (Fig. 2). This project relies on 4 pillars: surveillance; prevention and control; outbreak management; and community engagement. The Phase 1 field study implemented since October 2016 demonstrated that the released male *Wolbachia*-infected *Aedes* mosquitoes successfully competed with the urban male mosquitoes and were able to mate with the urban female mosquitoes. Most of the captured *Wolbachia* males were collected within a short distance from the release point but did show a good distribution throughout the area. As a result, the releases led to a 50% suppression of the urban *Ae. aegypti* mosquito population in the study sites. Since April 2018, NEA is conducting a Phase 2 study for improving the release methodologies to mitigate the problems presented by Singapore’s high density and high-rise urban landscape.

Professor Jeffrey Scott (Cornell University, USA) gave an overview of the challenges for controlling insecticide resistance in insect vectors of human diseases. In order to slow the evolution of resistance, two pieces of information are critically important: accurate assessment of the phenotype, and methods for the rapid determination of the frequency of the mutations that confer resistance in field populations. Resistance phenotype is not a binary trait and for assessing a phenotypic response in assays we need to look beyond evaluations based on a single diagnostic dose or concentration. There is also a need for a rapid and accurate assessment of the frequency of resistance mutations underlying the phenotypic response. This is somewhat simpler for mutations in target site genes, but is much more challenging for other major resistance mechanisms such as detoxification-mediated resistance. Identification of the mutations causing resistance and their fitness costs are critical to facilitate understanding of the evolution of resistance and to inform resistance management strategies.

During the ensuing general discussion, members of the audience raised concerns about the deliberate introduction of insecticide-resistant mosquitoes as a key component of the deployment of the *Wolbachia* technology [14]. Indeed, this may favor the survival of released mosquitoes in areas where insecticides are in common use and might contribute to the spread and homogenization of resistance in natural populations. Dr O’Neill stated that the *Wolbachia-*carrying mosquito strain was back-crossed with a local strain and that the resistance profile of released *Wolbachia*-mosquitoes simply matched that of the local “wild type”. Dr O’Neill encouraged the integration of routine monitoring of insecticide resistance in future efficacy trials with *Wolbachia*-carrying mosquitoes.

## Session 2: Insecticide resistance in arbovirus vectors

This session started with talks describing the status and spatial distribution of insecticide resistance in arbovirus vectors around the globe.

Dr Fara N. Raharimalala (Institut Pasteur, Madagascar) presented the insecticide susceptibility status and detoxifying enzymes activity in larvae and adults of *Aedes albopictus* in Madagascar. Mosquitoes were sampled in six localities (Antananarivo, Toamasina, Farafangana, Antsiranana, Mahajanga and Morondava) and then subjected to biological and biochemical assays. All mosquito larvae were resistant to temephos. Regarding adults, all mosquito populations were susceptible to fenitrothion and deltamethrin except those of Antananarivo and Mahajanga, respectively. Biochemical studies revealed an overproduction of detoxification enzymes (mainly esterases and cytochrome P450) that correlated well with phenotypic resistance. This study provides the first baseline information on insecticide resistance in *Ae. albopictus* in Madagascar. Further investigations are needed to address the genetic basis of insecticide resistance in field populations.

Dr Sébastien Marcombe (Institut Pasteur, Lao PDR) investigated the status, distribution and mechanisms of insecticide resistance in dengue vectors in Laos. Routine monitoring surveys conducted in 12 provinces showed moderate to high temephos resistance in *Ae. aegypti* and *Ae. albopictus*. Based on that finding, the National Strategic Plan 2019 for dengue control has been revised to stop using temephos and adopt a rotation scheme based on *Bti*, spinosad, and diflubenzuron. Adult bioassays showed resistance to malathion (organophosphate) and DDT (organochlorine) in *Ae. aegypti* and *Ae. albopictus. Aedes aegypti* also showed resistance to permethrin and deltamethrin. Biochemical assays showed higher activities of esterases and oxidases in natural populations compared to the susceptible USDA strain. Copy number variants (CNV) affecting the carboxylesterase CCEAE3A and the cytochromes P450 CYP6BB2 and CYP6P12 were detected by qPCR and were significantly correlated with insecticide resistance. In contrast, no clear association between the frequency of *kdr* mutations, for both 1534C and 1016G, and the mosquito survival rate to DDT and permethrin was observed. Altogether, these results demonstrate that metabolic-based resistance plays a major role in insecticide resistance in *Ae. aegypti* in Laos. These findings have important implications for dengue vector control and highlight the urgent need to identify new insecticides and innovative strategies to fight against arboviruses vectors.

Dr João Pinto (Instituto de Higiene e Medicina Tropical, Portugal) reported the origin and insecticide susceptibility status of a recently introduced *Ae. albopictus* population from Portugal. During the summer of 2017, two independent introduction events of the invasive mosquito *Ae. albopictus* were reported in Portugal from Hotel resorts located in Penafiel, Porto and Vilamoura, Faro. A preliminary analysis of 16 microsatellite loci suggest two independent origins for the introductions of *Ae. albopictus* in north and south of Portugal [15]. Bioassays carried out on the F1 generation showed full susceptibility of *Ae. albopictus* to permethrin (0.25%), deltamethrin (0.03%), cyfluthrin (0.15%), and fenitrothion (1%), and suspected resistance to bendiocarb (1%) according to WHO criteria [16]. It is important to continue mosquito surveillance and insecticide resistance monitoring to prevent the establishment and spread of invasive mosquitoes in Portugal.

Dr Ademir Martins (FIOCRUZ/IOC, Brazil) started by describing the Insecticide Resistance Monitoring Programme conducted in Brazil after the Zika outbreak. Previous monitoring studies (1999–2013) conducted in 102 sentinel municipalities have shown high resistance of *Ae. aegypti* to temephos and deltamethrin. Since then, the country reported more than 60 and 75% of probable cases of dengue and chikungunya, respectively in 2016, and more than 20% of Zika cases reported in the Americas so far. To guide decision making for vector control, the largest countrywide insecticide resistance monitoring (IRM) programme to date has been implemented by the dengue national control programme coordinated by the Ministry of Health (MoH). In total 146 municipalities were elected for sampling during 2017–2018, and the eggs shipped to two reference laboratories, where diagnostic dose bioassays with pyriproxyfen and malathion being performed, as well as *kdr* genotyping. The results will help the MoH to implement targeted chemical control of *Ae. aegypti* in the country.

Dr Nelson Grisales (Abt Associates, USA) described the Zika AIRS Project (ZAP) funded by USAID that aims to implement systematic insecticide resistance monitoring for *Aedes* mosquitoes in seven Latin American and Caribbean countries previously affected by Zika (i.e. Guatemala, Honduras, El Salvador, Paraguay, Guyana, Jamaica and the Dominican Republic). The ZAP builds systems, technical capacity, and promote appropriate resourcing in support of insecticide resistance testing in each country. After having reviewed the gaps and challenges for strengthening country capacity in entomological surveillance, the ZAP has developed a comprehensive approach to institutionalizing resistance testing according to the country needs that is (i) training of skilled staff, (ii) establishment of high quality entomology laboratories; and (iii) raising awareness on the importance of resistance testing. Although challenges remain, important elements are now in place to provide a foundation for sustained insecticide resistance testing in the region.

Dr David Weetman (Liverpool School of Tropical Medicine, UK) described the work performed by the WIN community to review available evidence on the spatial distribution of *Aedes* insecticide resistance and underlying mechanisms. About 6900 bioassay data points were collected and overall, 57 countries (87% of the total) showed confirmed or suspected resistance to at least one insecticide [4]. Resistance to all four main public health pesticides classes (pyrethroids, organophosphate, carbamates and organochlorines) is present in the Americas, Africa and Asia but distributions are not homogeneous, suggesting both challenges and opportunities for resistance management. Overexpression of resistance-associated detoxification enzymes appears widespread, and likely involves many genes. Estimating insecticide resistance is currently being challenged by a lack of standardization and diagnostic doses, but could be greatly assisted by calibration and predictive application of existing and novel DNA diagnostics for resistance. Widespread resistance calls for the careful use of existing formulations and implementation of insecticides with alternate modes of action.

The following presentations were dedicated to the understanding of resistance-associated molecular mechanisms; the evaluation of the impact of resistance on vector control; and the development of insecticide resistance management strategies for arbovirus vectors.

Dr Jean-Philippe David (Centre National de la Recherche Scientifique, Grenoble, France) presented the advantages of using an integrated approach combining experimental evolution, quantitative genetics and next-generation sequencing to identify novel genetic markers of insecticide resistance in the dengue mosquito *Ae. aegypti.* Whilst there are well-established markers for target-site mechanisms, reliable markers for metabolic resistance remain rare. Recently, deep targeted DNA-sequencing successfully identified several copy number variations (CNV) affecting cytochrome P450s (Cyp6 & Cyp9 families) that were associated with deltamethrin resistance [17]. The number of CNV was significantly correlated with increased gene expression levels obtained from RNA-seq [18]. Molecular investigations of *Ae. aegypti* samples from Laos showed that genomic amplification of an esterase cluster previously associated with temephos resistance in larvae was also strongly associated with adult resistance to malathion. These findings demonstrate that CNVs are promising DNA markers for tracking metabolic resistance because (i) they are frequent in *Ae. aegypti*, and (ii) they showed good association with resistance phenotype. These results pave the way for the development of novel diagnostic tools able to concomitantly track the whole range of insecticide resistance mechanisms in order to improve resistance monitoring and management.

Dr Shinji Kasai (National Institute of Infectious Diseases, Japan) described the first occurrence of the knockdown resistance (*kdr*) allele V1016G in *Ae. albopictus* in Asia and Europe. Overall, 30 *Ae. albopictus* populations were collected in Vietnam, Italy, Singapore, Brazil and Taiwan. Bioassays revealed that most populations of *Ae. albopictus* were highly susceptible to permethrin but a few from Italy, Vietnam and Singapore, exhibited resistance. Genotyping studies detected the *kdr* alleles F1534C in Vietnam and Singapore and F1534S in Vietnam and V1016G in samples from Vietnam and Italy for the first time in history [19]. Establishment of colonies homozygous for each *kdr* allele showed that 1016G allele caused much greater levels of pyrethroid resistance (5- to 13-fold) than 1534C or 1534S. The occurrence of the V1016G *kdr* mutation in the tiger mosquito represents a new threat to the control of this species worldwide.

Mrs Erly Sintya Dewi (Universitas Warmadewa, Indonesia) presented the status of insecticide resistance of *Ae. aegypti* in the Indonesian island of Bali and its implications for dengue control. WHO tube tests carried out on field-caught *Ae. aegypti* mosquitoes showed low mortality rates when exposed to diagnostic concentrations of permethrin (5% mortality), alpha-cypermethrin (14% mortality) and to a lesser extent, malathion (60% mortality). Mosquitoes surviving permethrin exposure exhibited higher frequencies of *kdr* S989P and V1016G alleles than those killed [20]. Genome-wide variation analyses showed a decrease of diversity around the VGSC gene locus, indicating a selective sweep. The use of “free-flight” tests in patchily treated rooms demonstrated that the Bali strain was far less affected by permethrin (48% mortality) than the susceptible Australian *Ae. aegypti* strain used as a reference (94% mortality). Under similar conditions, malathion killed 100% of both *Aedes* strains. This study demonstrates that insecticide resistance may compromise dengue vector operations relying on pyrethroids and the use of malathion represents the most pragmatic choice for the control of *Ae. aegypti* in Bali.

The presentation of Dr Gabriela Gonzalez-Olvera (Universidad Autónoma de Yucatán, Mexico) focused on the impact of household aerosolized insecticides on pyrethroid-resistant *Ae. aegypti.* Mismatch between the frequency of pyrethroid resistance in mosquitoes and the occurrence of pyrethroid-based insecticide applications for vector control has been observed in many places in Latin America [21] and could be due to the intense household use of commercial insecticide products. Through experimental assays quantifying phenotypic and genotypic responses of mosquitoes exposed to commonly used household aerosols, the authors showed significantly lower mortality rates (40–50%) of three pyrethroid-resistant field *Ae. aegypti* strains compared to the laboratory susceptible strain (99%). Applying insecticides as surface sprays led to a significant increase in the frequency of *kdr* V1016I homozygotes in surviving *Ae. aegypti*, suggesting strong selection pressure for this allele [22]. Given the large-scale use of household aerosol insecticide products in areas that are endemic for *Ae. aegypti*-transmitted diseases, their role in selecting pyrethroid resistance, should be taken into consideration when designing resistance management plans.

Dr Gregor Devine (QIMR Berghofer, Australia) talked about the problem of dispersion of invasive vectors facilitated by the global movement of people and cargo by aeroplanes and reviewed the WHO procedures for aircraft disinsection [23]. In Australia, disinsection procedures for aircraft entering the country are increasingly reliant on the residual treatment of cabins and holds with 200 mg/m^2^ permethrin applied at 8-week intervals. The impact of pyrethroid resistance on the efficacy of permethrin residual application has however never been explored. Through a series of bioassays conducted on a range of treated aircraft surfaces and highly permethrin-resistant *Ae. aegypti* strains (homozygous for 989P and 1016G), the author showed very poor efficacy of permethrin, particularly on carpets and seat covers (0–10% mortality). This was the result of insecticide resistance and the poor bioavailability of permethrin on absorptive surfaces (as confirmed by high-performance liquid chromatography, HPLC). The 24 h exposure of insecticide-resistant, free-flying mosquitoes to patchily-applied residues in a 20 m^3^ flight chamber resulted in < 25% of the mortality seen for insecticide susceptible mosquitoes. In contrast, malathion at 2 g/m^2^ was effective against those resistant strains, so alternative chemistries, although not registered for use on aircraft, may still be used “ground side” to protect passenger disembarkation and baggage handling areas. New disinsection chemistries and application methods are needed to protect Australian borders, communities and the insecticide-susceptibility of local endemic mosquito populations.

**Fig. 3:**
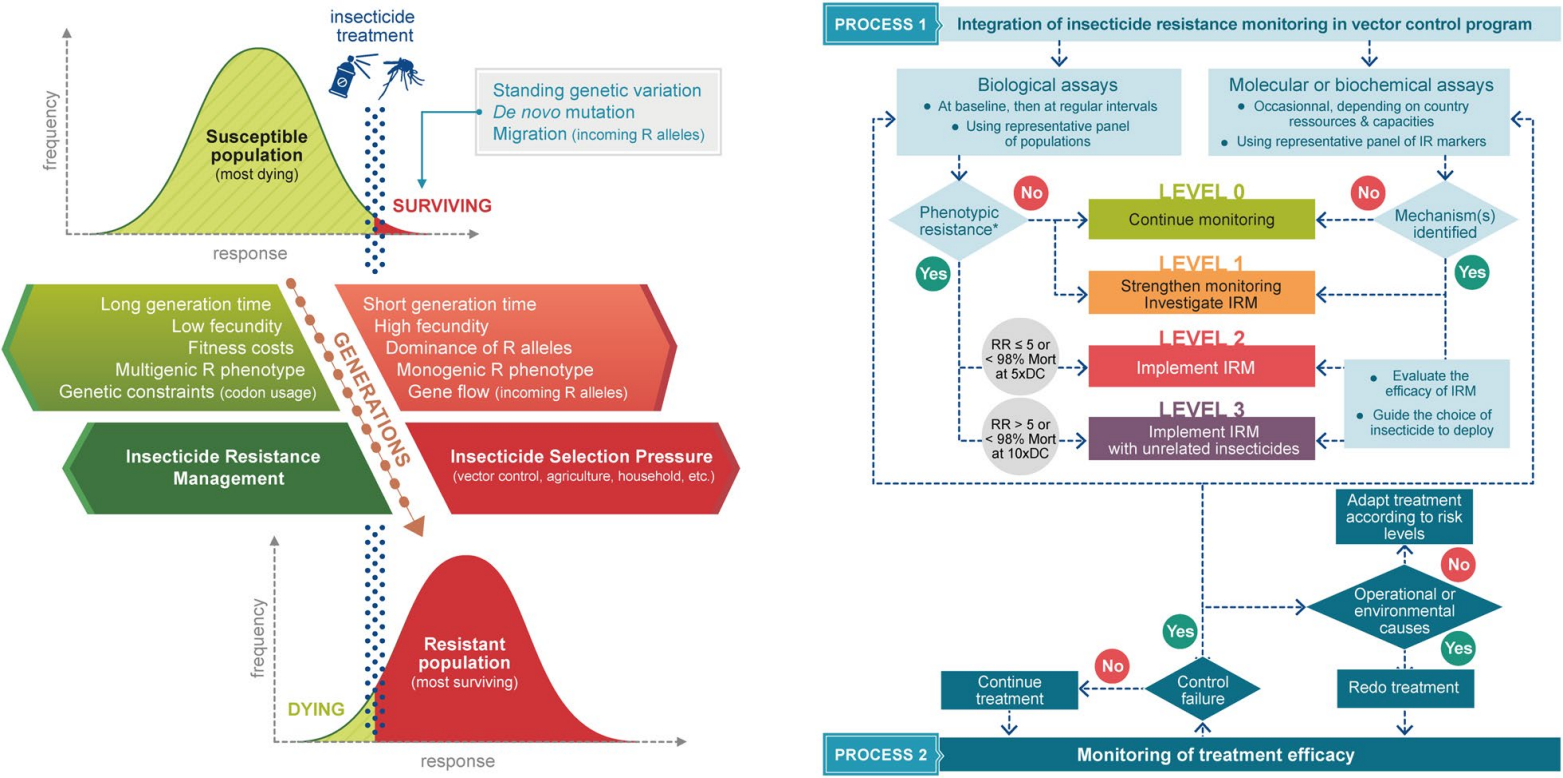
Management of insecticide resistance in *Aedes* vectors. Factors contributing to the selection of insecticide resistance in mosquitoes (left panel). Conceptual framework for implementing IRM in *Aedes* (right panel) (Copyright: Creative Commons Attribution 4.0 License (https://creativecommons.org/licenses/by/4.0/). Citation: Dusfour et al. (2019) Management of insecticide resistance in *Aedes* vectors: advances and challenges. PLoS Negl Trop Dis. 2019 (In Press) [24])

This session ended with a presentation from Dr Fabrice Chandre (Institut de Recherche pour le Développement, France) about insecticide resistance management (IRM) strategies applicable to mosquito vectors of arboviruses [24]. This work coordinated by the Worldwide Insecticide Resistance Network (WIN) aimed at defining the principles and concepts underlying IRM, identifying the main factors affecting the evolution of resistance and evaluating the value of existing tools for resistance monitoring (Fig. 3). Based on the lessons taken from resistance management strategies used for other vector species and agricultural pests, the speaker emphasized on the need for urgent action to contain insecticide resistance in invasive mosquitoes and proposed a roadmap for the implementation of a global plan for IRM in *Aedes* mosquitoes.

## Session 3: Innovative vector control approaches for emerging arboviruses

This morning plenary session started with five presentations on control strategies and new tools for improving *Aedes* control and surveillance.

Professor Dina Fonseca (Rutgers University, USA) opened this session by presenting a “success story” for the control of *Aedes* mosquitoes through a “Citizen Action through Science” (Citizen AcTS) approach that engages community members to perform mosquito control in their yards. This approach was tested in a NE US town of approximately 1000 residential yards infested with the invasive Asian tiger mosquito, *Ae. albopictus* [25]. After consulting with Rutgers entomologists, members of the community purchased, deployed and maintained 1032 Gravid *Aedes* Traps (GATs) two per yard out of 954 potential yards (46%) starting in June of 2017. To assess the effectiveness of the intervention during August and September a team from Rutgers deployed BG Sentinel traps in 19 yards across the town. They found that a GAT coverage higher than 80% in neighborhood clusters resulted in significant decreases in host-seeking female *Ae. albopictus*. This community-based approach works through respectful exchanges among scientists and residents that lead to trust and individual ‘buy-in’. Results of the surveys were quickly provided to the residents at the end of the season and have helped maintain the interest and enthusiasm.

The development of a new gravid trap for mosquito surveillance and control in Singapore was the central topic of the presentation by Dr Chee-Seng Chong (Environmental Health Institute, National Environment Agency (NEA), Singapore & Nanyang Technological University, Singapore). The Gravitrap is a black cylindrical contraption that contains aged hay infusion as a lure to attract female *Aedes* mosquitoes that are seeking water containers to lay their eggs. Gravitraps were placed in 2013 in 580 residential blocks within 34 sentinel locations to address the spatial dynamics of *Aedes* population [26]. After 5 years, 50,000 datapoints were recorded for resource prioritization. The results show that *Aedes* mosquitoes were heterogeneously distributed among blocks and among floors within the block. The abundance of *Ae. aegypti* was positively associated with the age of the blocks. A before-after-control-impact (BACI) analysis to compare the dengue-case ratio between estates with and without Gravitraps indicated a 30% reduction in case burden in estates with Gravitraps. Beyond providing spatial and temporal data on vector risk, the direct removal of the adult females by Gravitraps deployed in public housing has shown to have an epidemiological impact.

Dr Alongkot Ponlawat (Vector Biology & Control Section, Department of Entomology, USAMD-AFRIMS, Thailand) presented an overview of new vector control tools (VCTs) under investigation in Thailand. Laboratory experiments showed that blood-fed females of *Ae. aegypti* exposed to pyriproxyfen (PPF) had significantly less fecundity and fertility than unexposed females. Pre-exposure to PPF also reduced sperm production in males. A field trial involving 11 clusters in Muang District, Bangkok showed that clusters sprayed with the combination of pyrethrin and PPF (ULV or thermal fogging) had significantly lower numbers of *Ae. aegypti* 20 days post-application than the control (unsprayed cluster). These findings suggest that IGRs alone or combined with a pyrethroid adulticide may contribute to effective control of *Aedes* mosquitoes in Thailand.

Dr Sebastian Boyer (Institut Pasteur du Cambodge, Cambodia) presented results of a cluster randomized controlled trial aiming at evaluating an integrated vector control strategy (IVCS) targeting schools to prevent dengue and dengue-like syndrome (DLS). The trial was implemented in Kampong Cham Province, Cambodia with 24 clusters, 12 under integrated vector control and 12 without. Each cluster included one school, with an active surveillance of DLS in neighbouring villages (~15,000 children aged 5–15 years-old). The IVCS implied the removal of breeding sites in and around the school, the use of the bacterial insecticide *Bti* in permanent domestic water containers, deployment of In2care® traps for the dissemination of pyriproxyfen and spores of *Beauveria bassiana* and education and sensitization of children. Entomological preliminary data during the second year, following interventions, showed a 50% decrease in *Ae. aegypti* relative abundance in treated clusters compared to untreated clusters. Similarly, except for one school, there was a strong decrease in positive containers in all houses around the schools. Although epidemiological data acquisition is still in progress, first serological surveys showed fewer DLS in the treated (*n* = 485) than in the control cluster (*n* = 165), suggesting that IVM for dengue prevention worked well in schools.

**Fig. 4:**
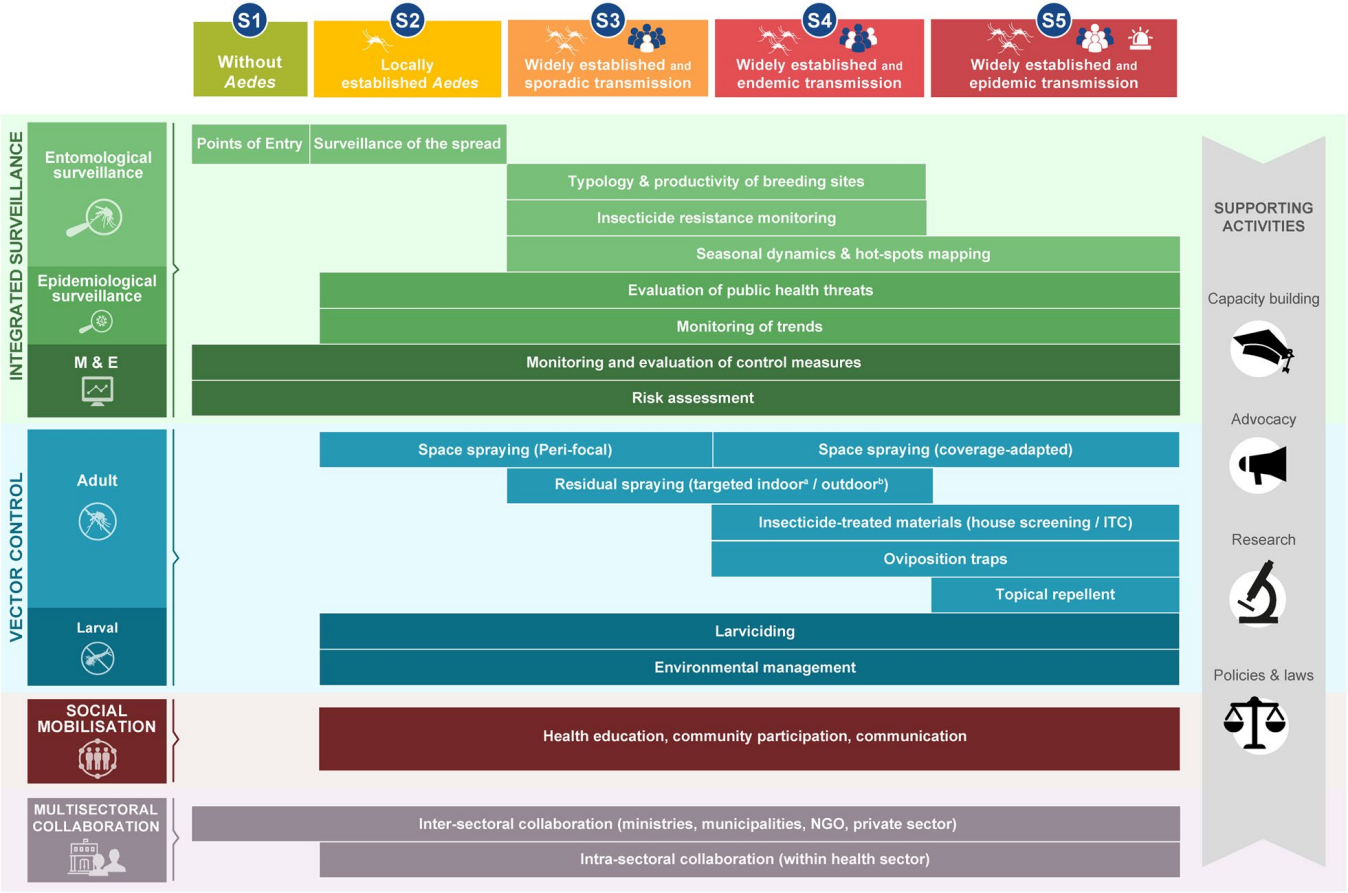
Conceptual framework of implementing an Integrated *Aedes* management for the control of *Aedes*-borne diseases (Copyright: Creative Commons Attribution 4.0 License (https://creativecommons.org/licenses/by/4.0/). Citation: Roiz et al. (2018) Integrated *Aedes* management for the control of *Aedes*-borne diseases. PLoS Negl Trop Dis. 2018;12:e0006845 [3])

Dr David Roiz (IRD, MIVEGEC, France) presented a framework for the implementation of an integrated *Aedes* management (IAM) for the control of *Aedes*-borne diseases [3]. IAM has been developed by the WIN network to provide national authorities with a comprehensive evidence-based guidance on how and when to implement *Aedes* control measures. IAM consists of a portfolio of operational actions and priorities for the control of *Aedes*-borne viruses that are tailored to different epidemiological and entomological risk scenarios. The framework has four activity pillars: (i) integrated vector and disease surveillance, (ii) vector control, (iii) community mobilisation, and (iv) intra- and intersectoral collaboration; and four supporting activities: (i) capacity building, (ii) research, (iii) advocacy, and (iv) policies and laws (Fig. 4). IAM supports the implementation of the WHO Global Vector Control Response that aims to devise and deliver sustainable, effective, integrated, community-based, locally adapted vector control strategies in order to reduce the burden of vector borne diseases worldwide.

Dr Nicole Achee (Notre Dame University, USA) gave a general overview of alternative strategies for mosquito-borne arbovirus control. The alternative strategies that have been presented reflect those that are currently under evaluation for public health value by WHO and various stakeholders and included novel larvicides/applications, spatial repellents, traps, attractive targeted sugar baits (ATSB), insecticide-treated materials, sterile insect technique (SIT), release of insects with dominant lethality (RIDL), *Wolbachia* and synthetic gene drive methods. Overall, the speaker described when and where these strategies/products may offer the greatest public health value [6]. Considerations to deployment, regulations, community acceptance, and sustainability were discussed. Although evidence is still lacking for most of these interventions, they may offer great potential for mitigating insecticide resistance, through an IVM approach, even if it is of a similar efficacy to existing interventions.

Dr Devi Shankar Suman (Ministry of Environment, India) gave an overview of insecticide-auto-dissemination technologies for mosquito control. The autodissemination strategy exploits the skip-oviposition behaviour of gravid females transferring small insecticide doses in an eco-friendly manner among breeding habitats [27, 28]. This approach is particularly interesting for *Aedes* mosquitoes that inhabit a wide range of artificial water containers and cryptic habitats in urban and suburban areas, that are difficult to control with conventional treatments [29]. The efficacy of locally made autodissemination stations using pyriproxyfen, an insect growth regulator and oviposition attractants were tested in residential areas infested with *Ae. albopictus* in New Jersey, USA [30]. The results showed that the stations effectively delivered pyriproxyfen in cryptic habitats where insecticides could not penetrate using conventional insecticide sprayers. Autodissemination stations significantly reduced the numbers of eggs, larvae and adult mosquitoes and hence represent promising alternative tool for the control of container-breeding mosquitoes.

Professor Gregor Devine (QIMR Berghofer, Australia) presented a talk on targeted indoor residual spraying (TIRS) for the control of *Ae. aegypti*. TIRS consists of spraying walls below 1.5 m and dark areas under furniture that are the favorite resting places for *Ae aegypti*. A retrospective study of public health GIS data from Cairns, Australia showed that contact tracing coupled with TIRS (lambdacyalothrin) around case residences and their potential exposure locations reduced the probability of future DENV transmission by 86–96%, compared to unsprayed premises [31]. The residual efficacy of conventional IRS against two TIRS methods using a carbamate insecticide against a pyrethroid-resistant, field-derived *Ae. aegypti* strain was further evaluated in Merida, Mexico. A clustered randomized control trial showed that TIRS and Resting-Site-IRS took 31% and 82% less time to apply, respectively, and used 38% and 85% less insecticide, respectively, than conventional IRS. The mortality of pyrethroid-resistant *Ae. aegypti* did not differ significantly among the three IRS application methods for up to two months post-application, and did not significantly differ between conventional IRS and TIRS up to four months post-application. These data illustrate that optimizing IRS to more efficiently target *Ae. aegypti* can both reduce application time and insecticide volume without reducing entomological efficacy.

Dr David Damiens (Institut de Recherche pour le Développement-CYROI, La Réunion Island, France) presented new developments in the use of the sterile insect technique (SIT) for *Ae. albopictus* control in La Réunion Island. From 2009 to 2014, the researchers developed a cost-effective adult holding cage for mass rearing that offers several advantages including weekly egg production of 250,000-400,000 eggs/cage, higher egg hatch rates and similar survival rate to the reference FAO/IAEA cage. Furthermore, they showed that irradiated sterile males demonstrated similar mating success as fertile males [32]. The second phase of the project will focus on field site characterization (in terms of spatial and temporal distribution of the mosquito population) and social mobilization and communication for the release of sterile mosquitoes at two pilot sites on the island.

**Fig. 5:**
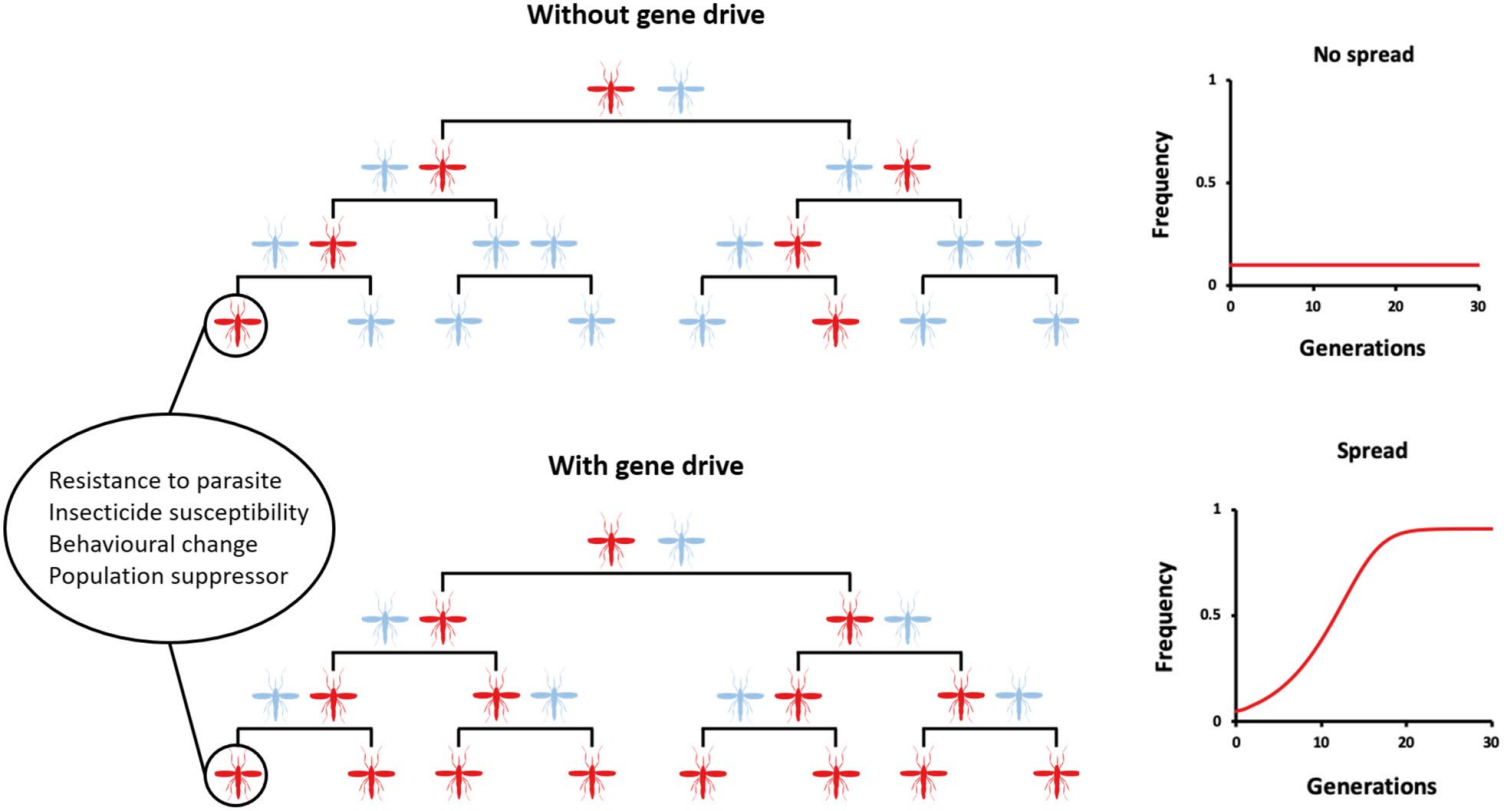
Gene drive inheritance concept. Gene drives copy themselves onto both chromosomes so the modified chromosome is inherited by all of the offspring (Courtesy of Mr Kyros Kyrou)

Mr Kyrou Kyros (Imperial College, London, UK) closed the session with a talk on gene drive technology for vector control. The recent development of CRISPR/Cas9 has unlocked the possibility to selectively edit a mosquito population with the goal of developing a novel vector control strategy (Fig. 5). Current genetic modifications designed to either impair female fertility or interfere with mosquito ability to transmit a malaria parasite have been shown to spread within large caged mosquito populations. In the laboratory, the team showed very strong transmission rates (up to 100%) into the progeny of three driven genes (AGAP005958, AGAP011377 and AGAP007280) which target female reproduction [33]. When AGAP007280 was tested in a population experiment, the spread proceeded as predicted for four generations but unfortunately, successive loss of the gene was reported from generation 8, hence indicating resistance to the drive. In contrast, a new CRISPR-Cas9 gene drive construct targeting the gene *doublesex* (*Agdsx*) of *An. gambiae* spread rapidly in caged mosquitoes, reaching 100% prevalence within 7–11 generations while progressively reducing egg production to the point of total population collapsed [34]. Given the conserved functional role of *dsx* for sex determination in all insect species and the high degree of sequence conservation amongst members of the same species, there is a potential of this gene drive system for targeting other vector species including *Aedes* taxa.

The session ended with an open discussion highlighting the promising results obtained in the development of new technologies and tools for mosquito control but acknowledging that evidence is still lacking to support their massive deployment by national control programmes. Questions were raised about the operational impact of gene drive technologies for controlling wild mosquito populations. Mr Kyros indicated that the potential of gene drive systems for field applications will be further evaluated as part of a phased approach in large confined spaces that mimic real ecological conditions more closely, in accordance with the recommendations of the USA American National Academy of Sciences.

## Session 4: Private/public initiatives to foster innovation in public health

This session aimed to discuss the challenges of insecticide resistance in the context of developing new tools, norms, and regulations for vector control. Representatives of the agrochemical sector from 12 companies, IVCC and other global initiatives (I2I, VectorBase, etc.) attended the conference to present on going activities, strategies and opportunities for improving vector-borne diseases control.

In the first session, five presentations were given by representatives of private companies that are involved in private-public partnerships for the development of innovative solutions for vector control.

Mr Peter DeChant (Valent BioSciences LLC, USA) presented IVM programmes relying on the use of *Bacillus thuringiensis* var. *israelensis* (Bti) strain AM65-52. Bti AM65-52 (Sumitomo Chemicals) has a unique mode of action, producing several cry toxins which in combination have high potential for resistance management [35]. Mr DeChant provided an overview of three observational studies conducted in Cambodia, Malaysia and Florida Keys, USA showing a strong impact of wide area application of Bti AM65-52 on the reduction of *Ae. aegypti* abundance and both dengue and Zika transmission [36–38]. Integration of Bti AM65-52 into operational programmes offer interesting prospects to prevent future outbreaks and to preserve the lifespan of current insecticidal chemistries, which are at-risk of operational failure due to resistance.

Mr Jason Nash (Bayer, Singapore) focused on Fludora Co-Max®, a new space spray combination for mosquito resistance management programmes. The rationale for developing this product was to look at active ingredients originally developed for agriculture that meet the requirements to be used in public health. This formulation combines two active ingredients with different modes of action (i.e. flupyradifurone, a butenolide, and transfluthrin, a pyrethroid) to enhance the control of insecticide resistance mosquitoes. Outdoor vehicle-mounted ULV spray in the USA and Brazil showed optimal control of insecticide-resistant *Aedes* mosquitoes (100% mortality) up to 100 m distance from spray origin. Fludora Co-Max® has a flexible use, being suitable for ULV, cold/hot fogging, and outdoor/indoor application and represents a promising technology for vector control and insecticide resistance management.

Mr Iñigo Garmendia (Goizper Spraying, Spain) started his presentation by providing the advantages of using IRS for the control of indoor biting/resting *Ae. aegypti* [39]. The efficacy of IRS actually depends on many operational factors including the quality of the spray. The speaker then described the performances of a new “iK vector control super sprayer” intended for indoor applications of insecticides. This new device has significant improvements over other devices, including the reduction of insecticide deposit variation on the wall through a constant nozzle flow rate, 50% reduction of insecticide loss, reduction of nozzles erosion over time, and reduction of contamination risk.

Mr Siao Jing Sam (Syngenta, Singapore) talked about Actellic 300CS, an organophosphate formulation for long-lasting IRS. This consists of an innovative microencapsulated formulation of pirimiphos-methyl (PM) that is expecting to deliver long-lasting residual control of mosquitoes (> 7 months) on porous surfaces. Several field studies are on-going in Africa to investigate the efficacy of Actellic 300CS in reducing mosquito biting rate and malaria transmission. Although some resistance to PM has been suspected in malaria vectors in some part of West Africa [40] Actellic 300CS has the potential for resistance management, either in rotation or mixture, with the aim to reduce the selection pressure by pyrethoids.

Dr James Austin (BASF, USA) addressed the potential of using non-repellent chemical insecticides for dengue management. Several solutions exist for dengue control and include indoor spraying, lethal oviposition traps, insecticide house screens, netting solutions, or applications of insecticides underneath furniture [41–45]. Chlorfenapyr (CFP), a repurposed insecticide from agriculture belonging to the pyrrole class has been evaluated in Australia for its potential use as an IRS. Phantom insecticide and Phantom pressurized insecticide have provided 100% control of *Ae. aegypti* mosquitoes in lab trials. Cone bioassay data 3 months after application of CFP at a rate of 250 and 500 mg/m^2^ on variable substrates showed 100% mortality of *Ae. aegypti* after 2-h exposure and 72-h holding period. Greater susceptibility of mosquitoes to CFP was observed when host-seeking and circadian rhythms were considered. Altogether these findings suggest that CFP is a promising chemical for *Aedes* control.

The second part of this session presented current initiatives and partnerships developed to foster innovation in vector control and resistance management as well as to accelerate a streamlined evaluation of pesticide products.

Dr Angus Spiers (Innovation to Impact, i2i, UK) discussed the actions that i2i launched in 2013 to promote innovation and accelerate the impact of new tools (https://innovationtoimpact.org/). Since the inception of i2i, this partnership has engaged a range of stakeholders encompassing the gamut of vector control partners to implement significant changes to the evaluation of vector control tools, most notably with the transition of WHO product evaluation from WHOPES to the WHO Prequalification Team (WHO-PQT) and the requirement for data to be produced at GLP certified sites. Currently 17 sites, 4 in Latin America, 6 in Asia, and 7 in Africa are included in the GLP accreditation process. i2i also tries to maximize impact at national level by speeding up country access to vector control tools and by minimizing the delay between WHO-PQT listing and registration by national regulatory authorities. Dr Spiers discussed remaining issues to be resolved to further optimize the evaluation and deployment of new vector control technologies such as lack of public health evidence for new tools, heterogeneity in regulatory pathways, and lack of quality control.

Mr Dominic Schuler (WHO-PQT, Switzerland) presented an update of the Prequalification Team for Vector Control (PQT-VC) at WHO (https://www.who.int/pq-vector-control/en/). WHO’s function for the evaluation of public health pesticides was transitioned from WHOPES to PQT in 2017 in order to harmonize approaches with the existing PQ product evaluation streams of medicines, vaccines, and diagnostics. The mandate of PQT-VC is to increase access to safe, high quality, efficacious vector control products. The first priority was to ensure vector control product “conversions” from WHOPES recommendations to PQT listing and to conduct inspections assessing the production facilities’ ability to produce vector control products. To date, 9 inspections have been conducted and 14 inspections are planned by the end of the 2019. PQT-VC currently works on label improvement to ensure that efficacy claims are supported by robust evidence and is evaluating 7 new submissions, including PBO LLINs.

Dr Nick Hamon (IVCC, UK) presented the development of a toolbox of solutions that can be deployed as part of an integrated vector management programme with the aim to control or even eliminate vector-borne diseases. IVCC is a product development partnership (PDP) that works with funders, innovators, academic groups, testing facilities, and international organizations to develop new solutions for vector control (http://www.ivcc.com/). IVCC has supported the development and launch of several new tools for tackling malaria vectors and has major collaborative projects with leading companies for the development of novel insecticide active ingredients for use in public health and the adoption of new resistance-breaking products through the IVCC NgenIRS programme funded by Unitaid. This programme, involving next generation products for indoor residual spraying, is now active in 18 African countries and is due to be followed up with a similar approach to support the introduction of novel LLINs. With the support of the Bill & Melinda Gates Foundation, DFID, USAID, DFAT, SDC and Unitaid, IVCC continues exploring a diverse range of emerging products and technologies for vector control, including *Aedes* vectors (e.g. electronic barriers, RNAi insecticides, “intelligent traps”, etc.).

Dr Florence Fouque (WHO-TDR, Switzerland) presented the legislative framework for vector control, with a focus on European countries. At the global level, countries which have agreed and signed the WHO International Health Regulations (IHR) document are recognizing some definitions and obligations on vectors of infectious agents constituting a public health risk, vector surveillance and control activities [46]. The absence of a harmonized legislative framework for implementation of vector-related activities at region or country level is posing problems not only for responding to emergency across borders, but also for testing and deploying new VCTs, such as traps, biological organisms and/or genetically modified organisms (GMO). For most EU countries, vector surveillance and control are under the Ministry of Health, but vector control products are under both Ministry of Health and Ministry of Environment, with authorizations based on EU legislation (Biocidal Products Directive 98/8EC). New vector control technologies using GMO, such as mosquitoes hosting *Wolbachia* sp. bacteria through transfection, have to be discussed under the rules of the Cartagena Protocol [47]. For most other countries, the tools using GMO do not have the adequate legislation to authorize large-scale testing. Consequently, there is a strong need for a global and harmonized legislative framework for vector-related activities.

Dr Samuel Rund (Notre Dame University, USA) described the PopBioMap by VectorBase.org, an online resource for insecticide resistance data that was developed by Notre Dame University, Imperial College London and EMBL-EBI with financial support from NIH (https://www.vectorbase.org/popbio/map/). The PopBioMap module has been created to respond to the emergence and spread of insecticide resistance in mosquitoes of public health importance. The PopBioMap is a graphical, map based, online tool for visualizing available information on the prevalence and mechanisms of insecticide resistance in vectors as well as surveillance data such as species, abundance, pathogen infections, etc. Data are submitted directly by researchers or extracted from publications by the VectorBase curators. The module contains significant amounts of genotypic and phenotypic data for major mosquito taxa (i.e. *Anopheles*, *Culex* and *Aedes*) helping national programmes to implement more effective, locally adapted vector control.

Dr Vincent Corbel (Institut de Recherche pour le Développement, France) closed this session by presenting the missions of the WIN. WIN is supported by the WHO Special Programme for Research and Training in Tropical Diseases (TDR) and the Department of Neglected Tropical Diseases (NTDs) since 2016 [7]. It brings together 19 internationally recognized institutions to track and combat insecticide resistance in arbovirus vectors worldwide (http://win-network.ird.fr/). Since its creation, the WIN has published 6 review papers to fill knowledge gaps on topics related to vector resistance and IVM and has organized biannual international conferences to foster innovation and strengthening mosquito control and surveillance efforts worldwide [8]. The network has gained international recognition for its role in mosquito resistance and is now expanding to a membership organization aiming at gathering all actors involved in vector-borne disease control (i.e. academia, international organizations, NGOs, not for profit organization, and the private sector). The ultimate goal of WIN is to build a global partnership to support international efforts to reduce the global burden of ABVs by 2030.

## Reports from Round Tables

### Round Table 1: Are the sustainable development goals (SDGs) for dengue and other arboviral diseases achievable with existing vector control tools?

The SDGs are a collection of 17 global goals set by the United Nations as part of Resolution 70/1 “Transforming our World: the 2030 Agenda for Sustainable Development” adopted in September 2015. The SDGs build on the success of the Millennium Development Goals (MDGs) and aim to go further to end all forms of poverty. Specifically, Target 3.3 aims to end the epidemics of AIDS, tuberculosis, malaria and neglected tropical diseases and combat hepatitis, water-borne diseases and other communicable diseases by 2030. Among communicable diseases, vector-borne diseases (VBDs) account for 17% of the global burden [2]. The WHO global vector control response, recently endorsed by member states, targets a reduction in mortality through VBDs by at least 75% by 2030. The question debated was “Are existing VCTs sufficient to reduce the burden of VBDs and especially ABVs and if not, what are the gaps that need to be addressed to achieve that goal?”

Workshop members agreed that examples of using existing VCTs to control ABV outbreaks do exist. These successes were predominately through operational impact using integrated approach of indoor and perifocal residual spraying, larval source reduction/treatment and social campaigns to reduce *Ae. aegypti* populations and dengue cases during the 1950s in Brazil, the 1970–1980s in Singapore and Cuba, among other examples. However, control failed primarily due to inability of systems to sustain these organized VC efforts [6].

Challenges to achieving success with existing VCTs continue and are dynamic. Evolving demographics (e.g. urbanization, lifestyles) and community/individual empowerment represent different conditions to that during the *Aedes* eradication era where VC implementation was largely dictatorial. WIN workshop participants acknowledged a general ‘resistance’ by populations and/or countries on chemical use that has facilitated maintenance of ABV exposure and therefore the risk of infection. Coverage remains a challenge for existing VCTs as not all larval and/or adult habitats can be easily accessed. Optimization of delivery systems may offer improvements to such coverage gaps. Similarly to existing tools, alternative VCTs will likely face many similar challenges. For example, implementation must be culturally appropriate and adopted for a strategy to have short-term and sustained impact—community-based approaches reflect this principle. There is no ‘one size fits all’ solution that an alternative VCT will resolve. In the same light, there is no ‘magic bullet’, a combination of tools, used in judicious and appropriate manner, the IVM concept, has proven to impact disease transmission most effectively also with new VCTs coming to the market. The WIN network has recently proposed a comprehensive framework for health authorities to devise and deliver sustainable, effective, integrated, community-based, locally adapted vector control strategies (IAM) in order to reduce the burden of *Aedes*-transmitted arboviruses [3].

Typically, an epidemic is over by the time vector control is initiated. The ability to prevent an ABV epidemic, and furthermore measures preventative impacts of an existing or alternative VCT requires precise and rigorous early-warning systems for both immatures and adults, implemented by vector control units. These units must have appropriate technical expertise and leadership. Participants noted that technical capacity is missing and that many ABV endemic countries may not take these responsibilities. Leveraging the experience of the malaria control units to apply to arbovirus control was deemed a viable approach to consider overcoming this gap; however, it remains at a core that political will and financial support is ultimately needed. A proposed “Global Fund” for arboviral diseases should be a discussion point across stakeholders in order to facilitate building in-country capacity to respond more effectively to these threats.

Workshop participants raised considerations regarding how to evaluate the ‘success’ of new VCTs. Specifically, it was mentioned that identification of appropriate endpoints for a VCT is critical to measure its impact. It is important to ensure that the evidence is made available to stakeholders in order to facilitate decision-making on procurement and use. It was recognized that WHO is faced with governments asking for evidence. Without evidence leaders cannot justify integrating a VCT into control programmes. For alternative VCTs where standard guidelines of efficacy testing are not available (e.g. SIT), such resources should be rapidly developed. Requirements are in place for large-scale epidemiological trials with randomized cluster trials (RCTs) to be considered the most informative studies [48]. RCTs are expensive to conduct but they are worth to generate the evidence needed to accelerate the deployment of new and effective VCTs. Where funding is limited, alternative study designs may be considered while accepting their limitations [48]. Modeling projections of impact was mentioned as a valuable component for study design development. Funding for epidemiological trials, regardless of design, must be forthcoming as without epidemiological evidence, WHO may not make recommendations for novel VCTs.

Although evidence is still lacking for most alternative strategies, they may offer great potential for mitigating insecticide resistance as part of an IVM approach through reduction of insecticide use, even if they are of a similar efficacy to existing interventions [6]. For example, optimizing IRS to more efficiently target *Ae. aegypti* can reduce both application time and insecticide volume without reducing entomological efficacy. It was acknowledged that public health is ‘starved’ for new active ingredients whereas agriculture is not, and that this issue needs to be solved. For those alternative VCTs that are chemical-based continued interest and investment in R&D through PPPs such as IVCC should continue to be advocated for repurposing agricultural chemistries, with novel modes of action, for public health purposes. To further incentivize investment, industry partners in PPPs need IP protection for first-in-class products.

Finally, there is a need to continue exploring a diverse range of emerging products and technologies for ABV vector control, particularly for *Aedes* spp. (e.g. acoustic larvicide, electronic barriers, RNAI insecticides, “intelligent traps”). The contribution of industry, foundations and international consortiums is essential for success. National legislation/regulatory framework will need to be adapted and/or be developed to address deployment of alternative VCTs with novel modes of action, without which evaluation and evidence-generation will be halted. These frameworks should be harmonized at the regional level to address cross-border concerns particularly where a new VCT such as the release of GM mosquitoes will have an impact beyond country borders. There is an urgent call for cross-sector coordination (i.e. multiple diseases, organizations, legislation) and continued financial support to achieve SDG 3.3.

### Round Table 2: Insecticide resistance: a trick or a real threat for vector control? Where is the proof that it is having an operational impact?

Insecticide resistance is an increasing challenge for *Aedes*-borne disease prevention because most dengue, Zika and chikungunya control strategies rely heavily on chemical control of the vector. Resistance or suspected resistance has been reported from at least 57 countries, including those in Southeast Asia, the Americas and the Caribbean where the dengue burden is particularly high [4]. Following renewed enthusiasm for strengthening vector control capacity, as witnessed at the May 2017 World Health Assembly [2], we need to assess whether those international efforts will be hindered by the presence and spread of resistance. Despite increasing concern, the degree to which insecticide resistance compromises *Aedes* control in the field remains largely unknown. Several entomological studies conducted in Latin America and the Caribbean show that insecticide resistance reduces the duration of efficacy for larval treatment [49, 50], the performances of pyrethroid space sprays and residual applications [51] and efficacy of household products [22, 52].

Further investigations are, however, needed to quantify the links between molecular insecticide resistance mechanisms, allele frequencies, resistance phenotypes and operational impact. That information would facilitate a pre-emptive risk assessment of control failure and improve the capacity of public health authorities to deploy or register products with greatest field efficacy. This endeavor is challenged by the fact that we lack the molecular tools required to identify, monitor and interpret anything other than a subset of resistance-associated mutations. As a consequence, we remain reliant on phenotypic studies; usually in the laboratory, but sometimes in the field, often augmented by the characterization of a small number of sodium channel mutations (known as kdr).

The purpose of Round Table 2 was to identify the impact of insecticide resistance on vector control operations and to identify related knowledge gaps. Participants in the discussion agreed that there are examples where the control of *Ae. aegypti* failed due to insecticide resistance (see references above), particularly for pyrethroids. Given that in most control programmes insecticide resistance is neither monitored nor evaluated, it is assumed that resistance-related control failures are under-reported and may be widespread. However, the group also recognizes that many control programmes do not have the resources to apply insecticides in an optimal manner leading to poor coverage, sub-standard operational practice (i.e. fogging outdoors in the heat of the day), and delayed responses [53]. There is no empirical evidence to link resistance-associated control failures to increased dengue, Zika or chikungunya transmission. However, until recently this has also been true for the more closely monitored and far better resourced malaria control programmes [54–57]. Studies specifically aiming to detect the epidemiological impacts of resistance are exceptionally hard to design, control and implement [58–60], especially for arboviral, urban diseases that show spatially and temporally heterogeneous transmission [61].

Accurate, affordable predictors based on entomological efficacy would be extremely useful for local authorities implementing public health measures including the procurement of insecticides. Such predictors might include “intensity assays” that compared to a single diagnostic dose may offer better information on the magnitude of resistance [62]. In addition, molecular assays to identify common resistance mutations are now simple and affordable enough to warrant adoption by a range of local authorities.

Current molecular assays for routine surveillance describe only a subset of mechanisms but may, at least for pyrethroids, provide useful “proxies” for incipient phenotypic resistance [63, 64]. For other chemical classes, molecular or biochemical tools are not yet available that reliably predict the resistance phenotypes. Well-designed, properly controlled field-trials, with entomological endpoints, especially those conducted against well-characterized phenotypes will help support the conclusions of more commonly applied bioassays and molecular diagnostics.

The value in testing and monitoring phenotypes and genotypes lies mostly in confirming the continued utility of existing chemistries. In the event that operationally relevant levels of resistance are encountered, there are few options for a change in practice. One might argue that, given the ubiquity and continued spread of pyrethroid resistance globally, all public health authorities should switch immediately to some kind of mosaic or rotation of pyrethroids, organophosphates, and carbamates to preserve mosquitoes susceptible. New chemical classes (e.g. butenolides, neonicotinoids) may help in this process as soon as they become available for large-scale deployment. We suspect that, once a chemical class is lost, it will be lost forever as resistance to pyrethroids has been found to be irreversible [65], although that may be due to continued use of pyrethroids in commercially available household aerosols [22].

For other potential elements of IRM and IVM strategies (e.g. untreated refuges, habitat management, biological control, late-life-acting insecticides) the evidence-base is limited and does not have the same universal relevance to control programmes as do insecticides. Community engagement will remain crucial, even if it is merely to increase acceptance and coverage of insecticide programmes. Apart from cost and complexity, the routine rotation of different chemical classes is complicated by shared issues of insecticide tendering and stockpiling.

## Summary and role for WIN

- Chemical insecticides remain the cornerstone of arbovirus vector control. In the medium term, there are no globally applicable alternatives. As a consequence, preserving the susceptibility to conventional insecticides should be the priority to all stakeholders and policy makers involved in vector borne disease control [66].
- When applied at high coverage, chemical-based interventions do have entomological impact, although epidemiological assessments of efficacy are rare [3]. Consequently, prioritisation of vector control strategies is difficult. The development of a “Global Fund” for ABVs would help to build in-country capacity to implement, monitor and evaluate interventions in order to generate the evidence require for decision making.
- Currently, it is hard to discriminate between vector control failures caused by sub-optimal use or by insecticide resistance. Regionally relevant trials, conducted against well-characterized IR mosquito populations, should be conducted to make an informed choice of intervention.
- Insecticide resistance is not binary, but rather continuous trait and lower levels of resistance may, temporarily, overcome by increased application rates. Accurate assessment of the phenotype and early detection of mutations that confer resistance can help to adjust vector control policies before operational consequences or intervention failures occur.
- Few operational teams or national authorities have the capacities to monitor insecticide resistance in routine. However, research institutions can provide support to local authorities by training public health officers, share laboratories, provide the expertise to design trials, and evaluate vector control interventions [3].
- All public health authorities tend to use single products until they fail. Unfortunately, the pipeline of new public health insecticides is very narrow, which means that older products cannot be removed and replaced with new ones when resistance is detected in a target mosquito population. WIN advocates for promoting routine substitutions/rotations between chemical classes that proved to be effective against the target species and/or deployment of non-chemical strategies [6], even at higher immediate cost in order to preserve susceptibility over the long term.
- WIN can help defining operational best practice, norms and guidance for IRM and develop Standard Operating Practices for monitoring and evaluation of IRM strategies.
- WIN can advocate for funding for training courses and regionally relevant trials as well as for the development of regulatory framework to promote the concept of susceptibility in vector control programmes.

## Conclusions

*Aedes*-borne viral diseases are rapidly spreading globally, causing increasing health and economic losses. Social, environmental, and demographic changes have facilitated the selection, spread and proliferation of viruses, vectors and resistant alleles into new areas [67] and has probably driven an increased use of insecticides by both households and public health authorities. As a consequence, the number of countries that have reported insecticide resistance in *Aedes* mosquitoes have dramatically increased in the last decade [4]. A striking example is the recent introduction of the V1016G kdr mutation conferring resistance to pyrethroids in *Ae. albopictus* in Europe for the first time in history [19]. Resistance is now recognized as a growing public health challenge threatening the global fight against vector borne diseases. Despite the development of a dengue vaccine, its limited efficacy and the lack of any vaccines or drugs for other ABVs such as Zika and chikungyunya means that insecticides will remain an essential part of *Aedes*-borne disease control programmes and outbreak responses. It is critical to preserve as long as we can the “lifespan” of new and existing molecules. As such, incentives and regulatory frameworks to support the concept of insecticide susceptibility of vectors as a “public good” should be considered [66]. Since 2016, the WIN has established a network of internationally recognized experts to improve the surveillance and control of insecticide resistance in vectors of emerging arboviruses. The missions are to raise awareness and mobilize resources for strengthening country capacity in resistance monitoring, stimulating research efforts, advise decision makers for resistance management, and strengthen public-private partnership to accelerate the deployment of integrated VCTs. The ultimate goal of WIN is to support international efforts to reduce the global burden of ABVs by 2030.

## Data Availability

All presentations for which speakers provided their consent are freely accessible on the conference website (https://WINSingapore2018.com).
